# Association Between Angiotensin-Converting Enzyme Inhibitors and Angiotensin II Receptor Blockers With Antivascular Endothelial Growth Factor-Related Proteinuria in Solid Tumors: A Systematic Review and Meta-Analysis

**DOI:** 10.7759/cureus.105120

**Published:** 2026-03-12

**Authors:** Ryuichi Ohta, Taichi Fujimori, Kaoru Tanaka, Hidetoshi Hayashi

**Affiliations:** 1 Community Care, Unnan City Hospital, Unnan, JPN; 2 Internal Medicine, Shimane University, Matsue, JPN; 3 Medical Oncology, Kindai University Faculty of Medicine, Sakai, JPN

**Keywords:** angiotensin-converting enzyme inhibitors, angiotensin receptor antagonists, general medicine, neoplasms, proteinuria, renal insufficiency, vascular endothelial growth factor inhibitors

## Abstract

Anti-vascular endothelial growth factor (VEGF) therapy is widely used in the treatment of solid malignancies but is frequently complicated by proteinuria, which can affect treatment continuity and patient quality of life. Although angiotensin-converting enzyme inhibitors (ACEi) and angiotensin II receptor blockers (ARBs) are commonly used to manage proteinuria in chronic kidney disease, their potential protective role against anti-VEGF-related proteinuria remains unclear. A systematic review and meta-analysis were conducted to evaluate the association between ACEi/ARB use and the risk of any-grade proteinuria in patients with solid tumors receiving anti-VEGF therapy. Comprehensive searches of PubMed, Embase, and Web of Science were performed for studies published between January 2000 and September 2025. Observational studies comparing ACEi/ARB users with patients receiving other antihypertensive agents were included. Patients not receiving antihypertensive therapy were excluded to minimize confounding by indication. Pooled risk ratios (RRs) were calculated using a random-effects model. Five retrospective observational studies were included. Across studies, ACEi/ARB use was associated with a significantly lower risk of any-grade proteinuria compared with other antihypertensive agents (pooled RR = 0.63; 95% confidence interval = 0.42-0.95), with moderate heterogeneity. Sensitivity analyses demonstrated consistent directionality of effect, supporting the robustness of the findings. ACEi/ARB use was associated with a reduced risk of any-grade proteinuria among patients receiving anti-VEGF therapy. Although limited by the observational nature of the evidence and geographic concentration of available studies, these findings suggest a potential renoprotective role of renin-angiotensin system inhibition in this setting. Prospective studies are warranted to confirm causality and clarify clinical implementation.

## Introduction and background

Solid malignancies remain a leading cause of morbidity and mortality worldwide, and systemic therapy plays a central role in their multidisciplinary management. Among available systemic treatments, therapies targeting vascular endothelial growth factor (VEGF) have become integral components of treatment strategies for various solid tumors, including colorectal, lung, gastric, ovarian, and renal cancers [1-3]. By inhibiting tumor angiogenesis, anti-VEGF agents have demonstrated clinical benefits across multiple disease settings and treatment lines, contributing substantially to improvements in oncological outcomes.

Despite their therapeutic efficacy, anti-VEGF therapies are associated with characteristic adverse events, among which proteinuria and renal dysfunction represent clinically significant complications [4,5]. VEGF plays a critical role in maintaining glomerular endothelial integrity, and its inhibition can lead to endothelial injury, podocyte dysfunction, and increased glomerular basement membrane permeability [6]. Consequently, patients receiving anti-VEGF therapy may develop a spectrum of renal manifestations ranging from asymptomatic proteinuria to nephrotic syndrome and severe kidney injury necessitating treatment interruption.

Renal adverse events related to anti-VEGF therapy have important clinical implications, as they may compromise treatment continuity and negatively affect patients’ prognosis and quality of life. In practice, the occurrence of proteinuria or renal dysfunction often prompts dose reduction, temporary suspension, or permanent discontinuation of anti-VEGF agents [7]. However, evidence-based strategies for the prevention or management of anti-VEGF-associated renal toxicity remain insufficient, and current clinical practice largely relies on empiric approaches and individual clinician experience.

Angiotensin-converting enzyme inhibitors (ACEi) and angiotensin II receptor blockers (ARBs) are widely used renoprotective agents in patients with chronic kidney disease and diabetic nephropathy, where they reduce proteinuria and slow the progression of renal dysfunction [8,9]. These effects are thought to be mediated through reductions in intraglomerular pressure, suppression of the renin-angiotensin system (RAS), antifibrotic properties, and improvement of endothelial function [9]. Given these mechanisms, ACE inhibitors and ARBs have been postulated as potential therapeutic options for mitigating renal injury associated with anti-VEGF therapy.

In recent years, several observational studies, retrospective analyses, and case-based reports have suggested that ACE inhibitors and ARBs may attenuate proteinuria and renal dysfunction induced by anti-VEGF agents [10,11]. Nevertheless, the available evidence remains fragmented, with substantial heterogeneity in study design, cancer types, anti-VEGF agents used, outcome definitions, and analytical approaches. As a result, the consistency, magnitude, and generalizability of the reported renoprotective effects remain unclear. To date, no comprehensive synthesis has systematically evaluated the effectiveness and safety of ACE inhibitors and ARBs in patients receiving anti-VEGF therapy for solid tumors.

Therefore, a systematic review of existing clinical evidence is warranted to clarify whether ACE inhibitors and ARBs mitigate anti-VEGF-associated renal toxicity, including proteinuria and renal function decline, and to assess their potential impact on treatment continuity. By synthesizing available data, this review aims to provide clinically relevant insights to support the management of renal adverse events, optimize the safe continuation of anti-VEGF therapy, and inform future research and guideline development in oncology supportive care.

## Review

Methods

Study Design

This study was conducted as a systematic review in accordance with the Preferred Reporting Items for Systematic Reviews and Meta-Analyses 2020 statement [12]. The review aimed to evaluate the renoprotective effects of ACEi and ARBs in patients with solid tumors receiving anti-VEGF therapy.

Protocol Registration

The International Prospective Register of Systematic Reviews has registered the review protocol. The registered number was CRD420251274588.

Search Strategy

A comprehensive literature search was conducted in PubMed, Embase, and Web of Science. The search strategy combined Medical Subject Headings and free-text terms related to solid tumors, anti-VEGF therapy, ACEi, ARBs, and renal outcomes.

Key search concepts included neoplasms or solid tumors; anti-VEGF agents or VEGF pathway inhibitors (e.g., bevacizumab, ramucirumab, and aflibercept); ACE inhibitors or ARBs; and renal outcomes such as proteinuria, renal dysfunction, or kidney injury.

Searches were limited to articles published between January 2000 and September 2025, corresponding to the period during which anti-VEGF therapies became clinically available. Only studies published in English were included. Gray literature, conference abstracts, editorials, and review articles were excluded.

The detailed search strategies for each database are provided in the Appendix. In addition, the reference lists of all included articles were manually screened to identify potentially relevant studies not captured by the electronic searches.

Study Selection

Eligible studies met the following criteria: 1) included patients with solid malignancies receiving anti-VEGF therapy, 2) evaluated the use of ACEi and/or ARBs as an exposure or intervention, 3) reported proteinuria of any grade as an outcome, defined according to study-specific criteria or standardized toxicity grading systems, and 4) employed observational study designs (prospective or retrospective cohort studies, case-control studies) or post hoc analyses of interventional trials. Any-grade proteinuria was selected as the primary outcome because it was the most consistently reported renal adverse event across the included studies. Severe proteinuria (grade ≥3) was reported inconsistently and occurred infrequently, precluding reliable pooled analysis.

Studies reporting additional renal outcomes, such as renal function parameters (e.g., estimated glomerular filtration rate or serum creatinine) or treatment interruption related to renal toxicity, were included only if data on proteinuria were available.

Studies focusing exclusively on hematologic malignancies, pediatric populations, or non-anti-VEGF therapies were excluded. Single case reports, conference abstracts, reviews, and editorials were also excluded.

Two reviewers independently screened titles and abstracts to identify potentially eligible studies. Full-text articles were subsequently assessed for eligibility. Discrepancies were resolved through discussion, and when consensus could not be reached, a third reviewer adjudicated.

Data Extraction and Synthesis

Data extraction was performed independently by two reviewers using a predefined standardized data collection form. Extracted information included study characteristics (authors, publication year, country, and study design), patient demographics, cancer type, details of anti-VEGF therapy, antihypertensive medication use, renal-related outcomes, follow-up duration, and reported effect estimates.

Regarding antihypertensive exposure, studies were reviewed in detail to identify patient groups receiving ACE inhibitors and/or ARBs alone, and those receiving antihypertensive agents other than ACE inhibitors or ARBs. For the purpose of comparative analysis, patients who did not receive any antihypertensive medication were excluded to minimize confounding related to baseline blood pressure status and the clinical indication for antihypertensive treatment.

The methodological quality of included observational studies was assessed using the Newcastle-Ottawa Scale (NOS) [13]. Any disagreements in data extraction or quality assessment were resolved by consensus.

Statistical Analysis

Where sufficient data were available, quantitative synthesis was conducted focusing on any-grade proteinuria as the primary outcome. Effect estimates, including risk ratios (RRs), odds ratios (ORs), or hazard ratios (HRs), were extracted directly from the original reports or calculated from available raw data when not explicitly provided.

In the present meta-analysis, any-grade proteinuria was treated as a cumulative incidence outcome within each study. When event counts were reported, RRs were calculated based on the proportion of patients who developed proteinuria during the observation period defined in each study. In studies reporting adjusted estimates such as ORs or HRs, the reported estimates were extracted and interpreted as approximations of relative risk.

Follow-up durations varied across studies (e.g., treatment cycles, fixed follow-up periods, or treatment duration). Therefore, pooled analyses were based on relative effect estimates rather than time-to-event synthesis. The pooled estimates should thus be interpreted as reflecting relative differences in the cumulative risk of proteinuria during each study's observation period rather than as time-adjusted hazard estimates.

For comparative analyses, data were synthesized by contrasting patients receiving ACE inhibitors and/or ARBs alone with those receiving antihypertensive agents other than ACE inhibitors or ARBs. Patients who did not receive any antihypertensive medication were excluded from pooled analyses to reduce confounding related to baseline blood pressure status and clinical indication for antihypertensive therapy.

Pooled analyses were performed using a random-effects model (DerSimonian and Laird method) to account for anticipated clinical and methodological heterogeneity across studies. Statistical heterogeneity was assessed using the I² statistic, with values greater than 50% considered indicative of substantial heterogeneity.

Prespecified subgroup analyses were planned according to cancer type, type of anti-VEGF agent, and class of RAS inhibitor (ACE inhibitor vs. ARB), when data permitted. Sensitivity analyses were conducted by excluding studies with lower methodological quality, as assessed by the NOS.

Publication bias was evaluated using funnel plots and Egger’s test when a sufficient number of studies were available. All statistical analyses were performed using Easy R (EZR; Jichi Medical University, Shimotsuke, Japan), a graphical user interface for R (R Foundation for Statistical Computing, Vienna, Austria) designed for biostatistical analysis [14].

Results

Study Selection

**Figure 1:**
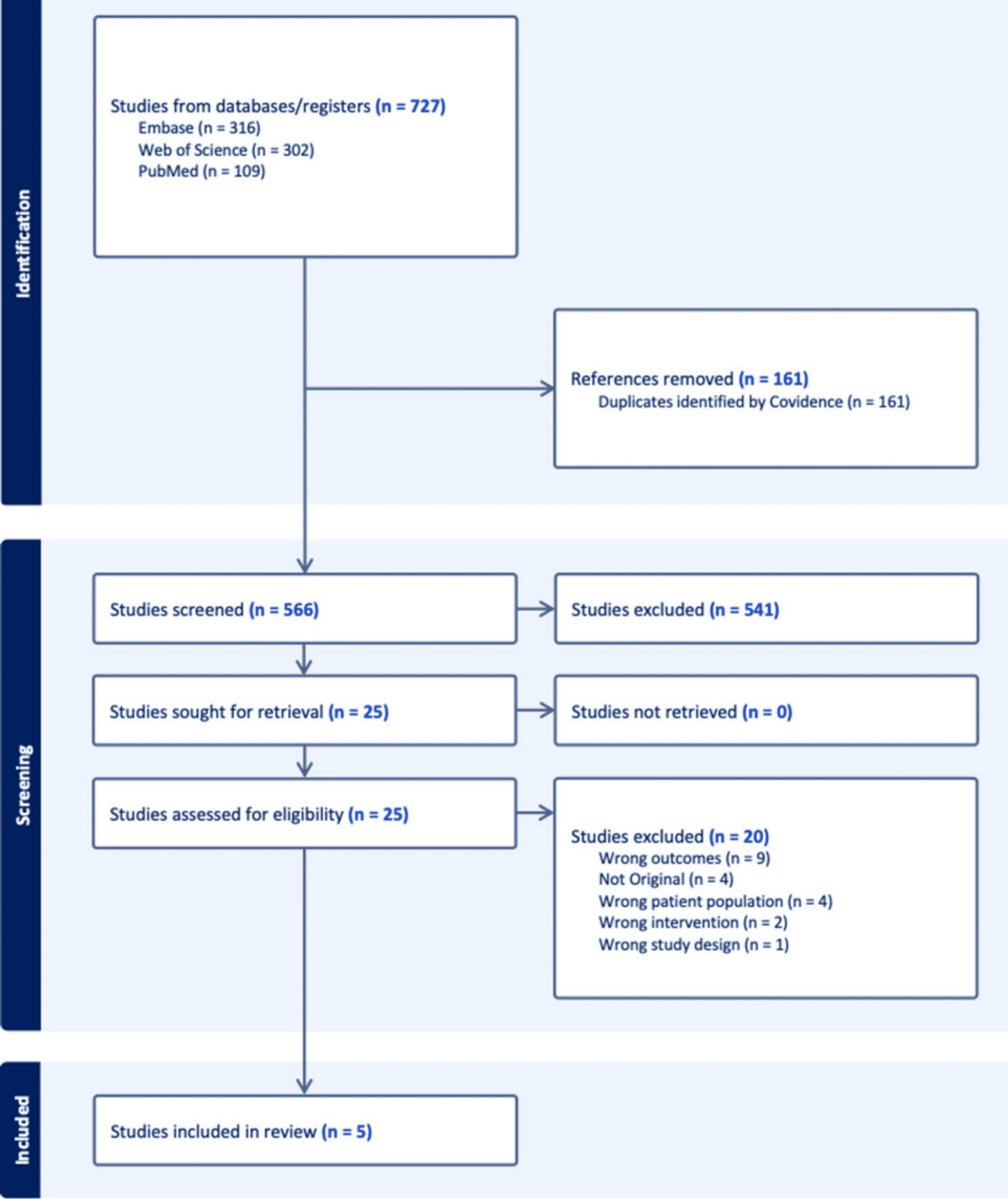
Selection flow

A total of 727 records were identified through database searches, including 316 from Embase, 302 from Web of Science, and 109 from PubMed. After removal of 161 duplicate records, 566 unique studies were screened based on titles and abstracts. Of these, 541 records were excluded during the initial screening. The remaining 25 studies were retrieved and assessed for full-text eligibility. Following full-text review, 20 studies were excluded for the following reasons: wrong outcomes (n = 9), not original research (n = 4), wrong patient population (n = 4), wrong intervention (n = 2), and wrong study design (n = 1). Ultimately, five studies met the inclusion criteria and were included in the systematic review. These studies reported data on any-grade proteinuria in patients with solid tumors receiving anti-VEGF therapy and allowed comparison between patients treated with ACE inhibitors and/or ARBs and those receiving other antihypertensive agents. Studies in which patients did not receive any antihypertensive medication were not included in the quantitative synthesis. The study selection process is summarized in Figure 1.

Characteristics of the Included Articles

A total of five observational studies were included in the systematic review. Notably, all included studies were conducted in Japan, reflecting the currently available clinical literature on this topic. All studies were conducted in adult patients with solid malignancies receiving anti-VEGF therapy and evaluated the association between ACEi and/or ARBs and the occurrence of any-grade proteinuria.

The included studies comprised retrospective cohort designs, with sample sizes ranging from small to moderate. Cancer types varied across studies and included colorectal, lung, gastric, renal, and other solid tumors. Anti-VEGF agents evaluated included monoclonal antibodies targeting VEGF or VEGF receptors as well as small-molecule VEGF receptor tyrosine kinase inhibitors.

Regarding antihypertensive exposure, all included studies provided data allowing comparison between patients treated with ACE inhibitors and/or ARBs alone and those treated with antihypertensive agents other than ACE inhibitors or ARBs. Patients who did not receive any antihypertensive medication were excluded from the comparative analyses.

Proteinuria was assessed using study-specific definitions or standardized toxicity grading systems, and all studies reported any-grade proteinuria as an outcome. Follow-up durations varied across studies, reflecting differences in treatment regimens and clinical settings. The characteristics of the included studies are summarized in Table 1.

**Table 1:** Characteristics of the included studies This table summarizes the key characteristics of the observational studies included in the systematic review. All studies were conducted in adult patients with solid malignancies receiving anti-VEGF therapy and evaluated the association between the use of ACEi and/or ARBs and the occurrence of any-grade proteinuria. Comparisons were restricted to patients treated with ACEi/ARBs and those treated with other antihypertensive agents; patients not receiving any antihypertensive medication were excluded. Anti-VEGF agents included monoclonal antibodies targeting VEGF or VEGF receptors as well as small-molecule VEGF receptor tyrosine kinase inhibitors. Proteinuria was assessed using study-specific definitions or standardized toxicity grading systems ACEi: angiotensin-converting enzyme inhibitor; ARB: angiotensin II receptor blocker; CRC: colorectal cancer; NSCLC: non-small cell lung cancer; TKI: tyrosine kinase inhibitor; VEGF: vascular endothelial growth factor; VEGFR: vascular endothelial growth factor receptor

Study	Country	Study design	Cancer type/population	Anti-VEGF agent	Anti-VEGF class	Follow-up
Nihei et al. [15]	Japan	Retrospective cohort	Nonsquamous NSCLC	Bevacizumab	Monoclonal antibody	Up to 6 cycles
Hirai et al. [16]	Japan	Retrospective cohort	Solid tumors (lung, gastric, colorectal, others)	Bevacizumab, ramucirumab	Monoclonal antibody	NR
Chiba et al. [17]	Japan	Retrospective cohort	Gastric cancer	Ramucirumab	Anti-VEGFR-2 monoclonal antibody	12 weeks
Ikesue et al. [18]	Japan	Retrospective cohort	Advanced/metastatic renal cell carcinoma	Axitinib	VEGFR TKI	Median treatment duration
Kiyomi et al. [19]	Japan	Retrospective cohort	Solid tumors (CRC, NSCLC, ovarian, breast, cervical)	Bevacizumab	Monoclonal antibody	12 months

Meta-Analysis of Any-Grade Proteinuria

A meta-analysis was conducted for the primary outcome, any-grade proteinuria, using data from five studies comparing patients receiving ACEi and/or ARBs with those in the control group. Using a random-effects model (DerSimonian and Laird method) implemented in EZR, ACEi/ARB use was associated with a significantly lower risk of any-grade proteinuria compared with control (pooled RR = 0.63, 95% CI = 0.42-0.95, p = 0.026). Because most included studies reported cumulative incidence rather than time-to-event data, the pooled estimates represent relative differences in cumulative risk during the study-specific observation periods.

**Figure 2:**
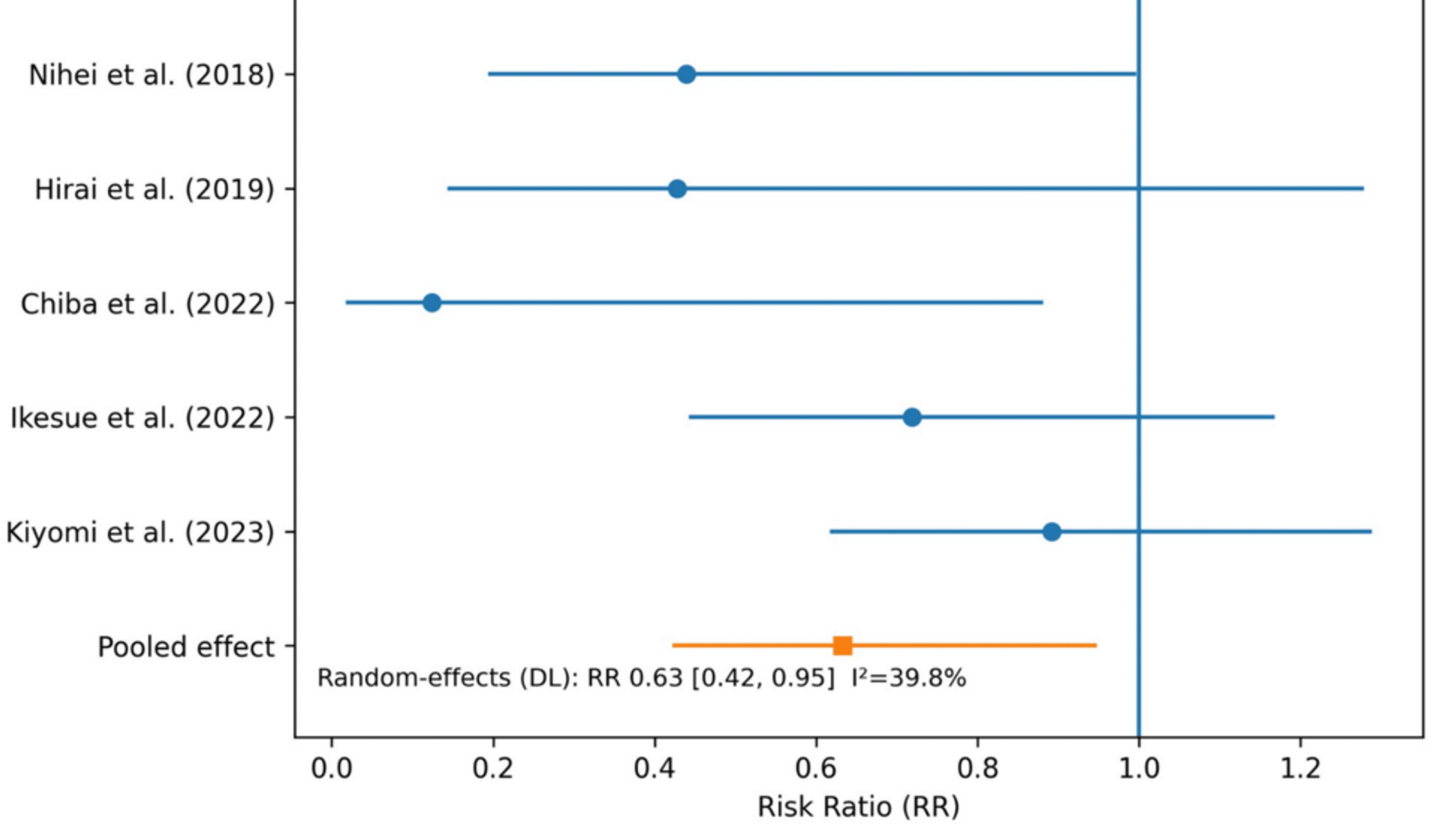
Forest plot of any-grade proteinuria comparing ACE inhibitors/angiotensin II receptor blockers with other antihypertensive agents. RRs and 95% CIs are shown for each included study (Nihei et al., 2018 [15]; Hirai et al., 2019 [16]; Chiba et al., 2022 [17]; Ikesue et al., 2022 [18]; Kiyomi et al., 2023 [19]), ordered chronologically by publication year. Squares represent study-specific effect estimates, with horizontal lines indicating 95% CIs. The vertical line indicates no difference between groups (RR = 1.0). The pooled estimate was calculated using a random-effects model (DL) RR: risk ratio; CI: confidence interval; DL: DerSimonian and Laird method

Between-study heterogeneity was moderate (I² = 39.8%). In leave-one-out sensitivity analyses, the direction of effect remained consistent (RR < 1 in all analyses), although statistical significance was attenuated in some iterations. Given the small number of included studies (n = 5), formal assessment of publication bias (e.g., funnel plot and Egger’s test) was not performed and should be interpreted with caution (Figure 2).

Sensitivity Analysis

To assess the robustness of the primary findings, leave-one-out sensitivity analyses were performed by sequentially excluding each study from the meta-analysis. Across these analyses, the pooled RRs for any-grade proteinuria remained consistently below unity, ranging from approximately 0.58 to 0.70, indicating a stable direction of effect favoring ACEi and/or ARB use.

Exclusion of individual large studies resulted in only modest changes in the pooled estimates and did not materially alter the overall findings. When the smallest study with the strongest individual effect estimate was excluded, the pooled effect size was attenuated, and statistical significance was lost, although the direction of association remained unchanged.

Overall, these sensitivity analyses suggest that the observed association between ACE inhibitor/ARB use and a reduced risk of any-grade proteinuria was not driven by a single study, supporting the robustness of the primary analysis.

Quality Assessment Results

The methodological quality of the included studies was assessed using the NOS for observational studies. Overall, the quality of the included studies was moderate to high. Most studies achieved favorable scores in the selection domain, reflecting an appropriate definition of study populations and clear ascertainment of exposure to anti-VEGF therapy and antihypertensive medications.

In the comparability domain, all studies adjusted for at least one relevant confounding factor, such as baseline blood pressure, renal function, or comorbid conditions. However, the extent and consistency of multivariable adjustment varied across studies, potentially leading to residual confounding.

In the outcome domain, proteinuria was assessed using either standardized toxicity grading systems or clearly defined study-specific criteria. Follow-up duration was generally sufficient to capture any-grade proteinuria during anti-VEGF therapy, although variability in monitoring frequency was observed.

No study was judged to be at high risk of bias across all domains. Nonetheless, limitations inherent to retrospective observational designs, including potential confounding by indication and incomplete adjustment for concomitant medications, should be considered when interpreting the results. The detailed results of the quality assessment are summarized in Table 2.

**Table 2:** Quality assessment of included studies using the NOS The NOS evaluates observational studies across three domains: selection of study groups (maximum 4 points), comparability of groups (maximum 2 points), and outcome assessment (maximum 3 points). Higher scores indicate better methodological quality NOS: Newcastle-Ottawa Scale

Study	Selection (max 4)	Comparability (max 2)	Outcome (max 3)	Total NOS score (max 9)
Nihei et al. [15]	3	1	3	7
Hirai et al. [16]	4	1	2	7
Chiba et al. [17]	3	1	2	6
Ikesue et al. [18]	4	2	3	9
Kiyomi et al. [19]	4	2	3	9

Discussion

Summary of the Study

In this systematic review and meta-analysis of observational studies, we evaluated the association between ACEi and/or ARBs and the risk of any-grade proteinuria among patients with solid tumors receiving anti-VEGF therapy. By restricting comparisons to patients receiving antihypertensive treatment, specifically ACE inhibitor/ARB users vs. users of other antihypertensive agents, and excluding patients without antihypertensive therapy, we aimed to reduce confounding from baseline blood pressure and treatment indication.

Across five studies, ACEi/ARB use was associated with a significantly reduced risk of any-grade proteinuria, with moderate heterogeneity across studies. Sensitivity analyses demonstrated consistent directionality of effect, supporting the robustness of the primary findings. Collectively, these results suggest a potential renoprotective role of RAS inhibition in the context of anti-VEGF-associated renal toxicity.

Comparison With Other Studies

Anti-VEGF-related proteinuria is a well-recognized adverse effect of VEGF pathway inhibition and is primarily attributed to glomerular endothelial dysfunction, disruption of the glomerular filtration barrier, and podocyte injury [5,8,20]. VEGF signaling plays a critical role in maintaining the structural and functional integrity of glomerular endothelial cells and podocytes, and its inhibition can lead to endothelial swelling, loss of fenestrations, and altered podocyte-endothelial crosstalk, ultimately increasing glomerular permeability [21]. Previous studies have mainly focused on the incidence, severity, and clinical management of proteinuria during anti-VEGF therapy, whereas preventive or protective strategies have received relatively limited attention [22,23].

RAS inhibitors, including ACE inhibitors and ARBs, are well established in the management of proteinuria in chronic kidney disease and diabetic nephropathy through their effects on reducing intraglomerular pressure, improving endothelial function, and stabilizing podocyte architecture [24]. Experimental and clinical data suggest that angiotensin II contributes to podocyte injury, oxidative stress, and inflammatory signaling within the glomerulus [25]. In the setting of VEGF inhibition, where endothelial injury and podocyte stress are already present, RAS activation may further exacerbate glomerular damage [26]. Thus, blockade of the RAS may attenuate proteinuria by mitigating downstream pathophysiological processes, including reduced efferent arteriolar vasoconstriction, reduced mechanical stress on the glomerular capillary wall, and preserved slit diaphragm integrity.

An additional consideration is the role of blood pressure control. ACEi and ARBs reduce systemic blood pressure, which may, in turn, lower intraglomerular pressure and reduce the risk of proteinuria. Therefore, the observed association between ACEi/ARB use and reduced proteinuria may partly reflect improved blood pressure control rather than intrinsic renoprotective mechanisms alone [24]. Because most included studies did not provide detailed longitudinal blood pressure data, the relative contribution of blood pressure-mediated effects vs. direct RAS-mediated renal protection could not be evaluated in this analysis.

Our findings extend the existing literature by quantitatively synthesizing available evidence and demonstrating a consistent association between ACE inhibitor/ARB use and a lower risk of any-grade proteinuria across heterogeneous cancer types and anti-VEGF agents. Importantly, by using other antihypertensive users as the comparator, our analysis minimizes bias introduced by comparing treated vs. untreated populations, a limitation present in some prior reports. The observed association is, therefore, not only clinically consistent with prior observational findings but also biologically plausible, given the complementary roles of VEGF signaling and the RAS in maintaining glomerular homeostasis and the potential for RAS inhibition to counterbalance VEGF inhibitor-induced renal injury.

Strengths of the Study

This study has several strengths. First, we focused on any-grade proteinuria, a clinically relevant outcome that often precedes severe renal toxicity and can influence treatment continuation and quality of life. Second, the comparator strategy, ACEi/ARB users vs. users of other antihypertensive agents, was deliberately chosen to reduce confounding by indication related to hypertension and cardiovascular comorbidity. Third, we adhered to rigorous systematic review methodology, including duplicate screening, standardized data extraction, quality assessment using the NOS, and random-effects meta-analysis. Finally, sensitivity analyses confirmed that the overall findings were not driven by a single study, reinforcing the stability of the observed association.

Limitations

Several limitations should be acknowledged. All included studies were retrospective observational cohorts, which preclude causal inference and leave residual confounding as a possibility. Although we restricted comparisons to patients receiving antihypertensive therapy in an attempt to reduce confounding by indication, systematic differences between ACE inhibitor/ARB users and users of other antihypertensive agents may still exist. For example, ACEi and ARBs are frequently prescribed to patients with underlying renal risk, diabetes, or cardiovascular disease, and prescribing decisions may also reflect clinician preference or perceived patient risk. Conversely, physicians may avoid these agents in patients with advanced renal dysfunction or other contraindications. As a result, differences in baseline renal risk, comorbidity burden, cancer severity, or other clinical characteristics may have influenced both treatment selection and the risk of proteinuria. Therefore, the observed association should be interpreted as an observational relationship rather than evidence of a causal renoprotective effect. Definitions and monitoring strategies for proteinuria varied across studies, potentially contributing to heterogeneity. In addition, detailed information on ACEi/ARB dose, duration, timing of initiation relative to anti-VEGF therapy, and medication adherence was inconsistently reported. Notably, all included studies were conducted in Japan, reflecting the available literature but potentially limiting the generalizability of the findings to other populations, healthcare systems, and prescribing practices.

Furthermore, although ACE inhibitors and ARBs are antihypertensive agents, the extent to which blood pressure reduction itself contributed to the observed decrease in proteinuria could not be adequately evaluated. Most studies did not provide detailed longitudinal blood pressure data, making it difficult to disentangle the direct renoprotective effects of RAS inhibition from the indirect effects mediated by blood pressure lowering.

The relatively small number of included studies limited the statistical power for subgroup analyses by cancer type or specific anti-VEGF agent and precluded a reliable assessment of publication bias. In addition, follow-up durations differed across the included studies. Because HRs were not consistently reported, the meta-analysis synthesized cumulative incidence measures rather than time-to-event outcomes. Differences in observation time may, therefore, have influenced absolute event rates, although the use of relative effect estimates partially mitigates this limitation.

Although severe proteinuria (grade ≥3) is clinically important and may lead to treatment discontinuation, reporting of high-grade events was inconsistent across studies, and the number of events was small. Consequently, quantitative synthesis for severe proteinuria was not feasible. Future studies with standardized reporting of high-grade renal toxicity are needed to better evaluate the impact of ACEi/ARB therapy on clinically significant proteinuria.

The geographic concentration of the included studies is also a limitation of the study. All eligible studies were conducted in Japan, which may limit the generalizability of the findings to other healthcare systems. Prescribing patterns for antihypertensive agents, blood pressure management strategies, and dosing practices for anti-VEGF agents may differ across countries and clinical settings. In addition, differences in patient characteristics, proteinuria monitoring practices, and healthcare infrastructure could influence both the detection and management of anti-VEGF-related renal toxicity. Future studies from diverse geographic regions are needed to confirm whether the observed renoprotective association of ACE inhibitors and ARBs is consistent across different populations and clinical environments.

## Conclusions

This systematic review and meta-analysis suggests that the use of ACEi and/or ARBs is associated with a lower risk of any-grade proteinuria in patients with solid tumors receiving anti-VEGF therapy compared with other antihypertensive agents. However, because ACEi and ARBs lower systemic blood pressure and may also exert direct renoprotective effects through renin-angiotensin system inhibition, the relative contribution of blood pressure control vs. intrinsic renal protective mechanisms cannot be determined from the available observational evidence.

## Appendices

Detailed literature search strategy

A comprehensive literature search was conducted to identify studies evaluating the association between angiotensin-converting enzyme inhibitors (ACEi) and angiotensin II receptor blockers (ARBs) and renal outcomes, particularly proteinuria, in patients with solid tumors receiving anti-vascular endothelial growth factor (VEGF) therapy. The search strategy combined controlled vocabulary (Medical Subject Headings (MeSH) and Emtree) and free-text keywords related to four core concepts: 1) solid malignancies, 2) anti-VEGF therapy, 3) renin-angiotensin system inhibitors, and 4) renal outcomes.

Electronic database searches were performed in PubMed, Embase, and Web of Science. The search period was restricted from January 1, 2000, to September 30, 2025, corresponding to the clinical introduction and widespread use of anti-VEGF therapies. Only studies published in English and involving human subjects were considered. Gray literature, conference abstracts, editorials, letters, and review articles were excluded to ensure the inclusion of original research with extractable data.

The final search strategies for each database were as follows.

PubMed (MEDLINE)

The PubMed search strategy combined MeSH and free-text terms:

("Neoplasms"[Mesh] OR cancer* OR tumor* OR tumour* OR malignan* OR "solid tumor*" OR "solid tumour*") AND ("Vascular Endothelial Growth Factor A"[Mesh] OR "Angiogenesis Inhibitors"[Mesh] OR bevacizumab OR ramucirumab OR aflibercept OR "anti-VEGF" OR "VEGF inhibitor*" OR "VEGF pathway inhibitor*") AND ("Angiotensin-Converting Enzyme Inhibitors"[Mesh] OR "Angiotensin Receptor Antagonists"[Mesh] OR "ACE inhibitor*" OR ACEi OR "angiotensin receptor blocker*" OR ARB*) AND ("Proteinuria"[Mesh] OR proteinuria OR "renal dysfunction" OR "kidney injury" OR "renal impairment" OR nephrotoxicity)

Filters applied: English language; publication date from January 1, 2000 to September 30, 2025; humans.

Embase

The Embase search was conducted using Emtree terms and title/abstract keywords:

('neoplasm'/exp OR cancer*:ti,ab OR tumor*:ti,ab OR tumour*:ti,ab OR malignan*:ti,ab OR 'solid tumor*':ti,ab) AND ('vascular endothelial growth factor'/exp OR 'angiogenesis inhibitor'/exp OR bevacizumab:ti,ab OR ramucirumab:ti,ab OR aflibercept:ti,ab OR 'anti-vegf':ti,ab OR 'vegf inhibitor*':ti,ab) AND ('angiotensin converting enzyme inhibitor'/exp OR 'angiotensin receptor antagonist'/exp OR 'ace inhibitor*':ti,ab OR acei:ti,ab OR 'angiotensin receptor blocker*':ti,ab OR arb*:ti,ab) AND ('proteinuria'/exp OR proteinuria:ti,ab OR 'renal dysfunction':ti,ab OR 'kidney injury':ti,ab OR 'renal impairment':ti,ab OR nephrotoxicity:ti,ab)

Limits applied: human studies, English language, publication years 2000-2025.

Web of Science Core Collection

The Web of Science search was performed using topic fields (title, abstract, and keywords):

TS = ((cancer* OR tumor* OR tumour* OR malignan* OR "solid tumor*" OR "solid tumour*") AND (bevacizumab OR ramucirumab OR aflibercept OR "anti-VEGF" OR "VEGF inhibitor*" OR "VEGF pathway inhibitor*") AND ("ACE inhibitor*" OR ACEi OR "angiotensin receptor blocker*" OR ARB*) AND (proteinuria OR "renal dysfunction" OR "kidney injury" OR "renal impairment" OR nephrotoxicity))

Refinements: document type = Article; language = English; timespan = 2000-2025.

In addition to electronic database searches, the reference lists of all included studies and relevant review articles were manually screened to identify potentially eligible studies that were not captured through the database search. Duplicate records were removed prior to title and abstract screening.
